# *Pristomyrmex tsujii* sp. n. and *P. mandibularis* Mann (Hymenoptera, Formicidae) from Fiji

**DOI:** 10.3897/zookeys.340.5479

**Published:** 2013-10-04

**Authors:** Eli M. Sarnat, Evan P. Economo

**Affiliations:** 1 Antwork Consulting, P.O. Box 563 Happy Camp, CA 96039 USA; 2 Okinawa Institute of Science and Technology Graduate University 1919-1 Tancha, Onna-son, Japan 904-0495

**Keywords:** Myrmicinae, *Pristomyrmex*, Fiji, taxonomy, ergatoid, islands, Pacific, new species

## Abstract

*Pristomyrmex tsujii* **sp. n**., an endemic species of the Fiji islands, is described from the worker, ergatoid queen, alate queen and male castes. The alate queen and male castes of *Pristomyrmex mandibularis* Mann are also described for the first time. The ergatoid queens for both species appear to be morphologically intermediate between the worker and alate queen castes. *Pristomyrmex tsujii* is readily distinguished from *Pristomyrmex mandibularis* by the lack of well-developed propodeal spines. Although both species occur across the Fijian archipelago, they are rarely encountered and workers are most often collected from sifted litter. The descriptions are illustrated with specimen photographs, line drawings and a distribution map.

## Introduction

*Pristomyrmex* Mayr is a genus composed of 59 extant valid species restricted to the Old World tropics (Bolton 2013). Its center of diversity is the Oriental region, though species are also known from the Australian rainforests, Africa, Mauritius and Reunion. Most species inhabit the rainforest, forage as predators or scavengers, and tend to nest in soil, leaf litter or rotten wood. A comprehensive and well-illustrated taxonomic revision of the genus across its range was completed by Wang (2003). Subsequent taxonomic work on the genus includes a review of the Philippine *Pristomyrmex* with three new descriptions and a key to species (Zettel 2006), and a key to the Taiwan species (Terayama 2009).

Fiji is the eastern range limit for *Pristomyrmex*, aside from *Pristomyrmex minusculus* Wang which is known from Tonga in addition toPapua New Guinea, Micronesia, and Queensland, Australia. *Pristomyrmex mandibularis* was first described by Mann (1921), and a second species from Fiji, described here as *Pristomyrmex tsujii*, was reported in Sarnat and Economo (2012) as *Pristomyrmex* sp. FJ02. Both species belong to the *levigatus* group. These ants are most often encountered in the leaf litter, and small colonies of *Pristomyrmex mandibularis* can be found nesting in rotting logs and under stones.

Both Fijian species produce a discrete ergatoid queen caste that is intermediate between a worker and an alate queen. Ergatoid queens in the genus have also been reported for *Pristomyrmex punctatus* Mayr, *Pristomyrmex africanus* Karavaiev, *Pristomyrmex brevispinosus* Emery and *Pristomyrmex wheeleri* Taylor. Previous studies (Heinze 1998; Peeters 1991) proposed that in truly ergatoid species the genetically winged female reproductive caste has been completely replaced by a permanently wingless female reproductive caste. By contrast, intermorph queens are a regularly produced caste that retain all reproductive functions of alate queens and can also co-occur with alate queens. Although the latter condition more accurately applies to both species of the Fijian *Pristomyrmex*, we continue to use the term ergatoid to describe the permanently wingless queen caste as it is broadly accepted across myrmecology and more easily facilitates comparative studies (Peeters 2012).

This taxonomic treatment of *Pristomyrmex tsujii* and *Pristomyrmex mandibularis* is part of an ongoing effort to characterize the systematics, ecology and evolution of the Fijian ant fauna (Economo and Sarnat 2012; Lucky and Sarnat 2008, 2010; Sarnat 2006, 2008; Sarnat and Economo 2012; Sarnat and Moreau 2011).

## Sources of material

All of the material examined in this study was collected from 2002–2007, including specimens collected as part of the Fiji Terrestrial Arthropod Survey (Evenhuis and Bickel 2005; Sarnat and Economo 2012) and in other collections made by the authors. All type material designated here will be deposited in the BPBM (Bernice Pauahi Bishop Museum, Honolulu, Hawaii, USA). Non-type material will be deposited in the ANIC (Australian National Insect Collection, CSIRO, Canberra, Australia), MCZC (Museum of Comparative Zoology Collection, Cambridge, MA, USA), and NMNH (National Museum of Natural History, Washington D.C., USA). Additional images along with additional specimen, collection and locality data for *Pristomyrmex tsujii* and *Pristomyrmex mandibularis* are available on Antweb.org. Geographic coordinates presented in the material examined sections are given in decimal degrees, rounded to the fourth significant digit where accuracy permits.

## Methods and abbreviations

External morphological characters were quantified and are reported as lengths or indices. Measurements were made with a stereomicroscope at 40x magnification using a dual-axis stage micrometer wired to digital readouts. Digital color specimen photographs were taken using the Auto-Montage software package (Syncroscopy) in combination with a JVC KY-F7U digital camera mounted on a Leica MZ16 dissecting scope, and the software package Helicon Focus in combination with a Leica DFC450 digital camera mounted on a Leica M205C dissecting scope. Vector illustrations were made in Adobe Illustrator by tracing specimen photographs.

Morphometric measurements were recorded in thousandths of millimetres, but are reported here to the nearest hundredth as a range from minimum to maximum across all measured specimens. Specimens for measurements were chosen to reflect potential morphological variation across the full geographic range. The number of specimens from which measurements were taken for a given caste is referred to by *n*. The following measurements and indices are adapted from Wang (2003).

CI Cephalic Index: HW/HL × 100.

EL Maximum measureable eye length.

HL Maximum length of the head in full-face view measured longitudinally from the midpoint of the posterior head margin to the apex of the median tooth of the anterior clypeal margin.

HW Maximum width of the head in full-face view, excluding the eyes.

HWE Maximum width of the head in full-face view, including the eyes. This measurement is used only for the male.

ML Mesosomal length measured in lateral view from the anteriormost point of the pronotum to the apex of the propodeal lobe. Equal to ‘Alitrunk Length’ in Wang (2003).

PeH Maximum height of the petiole measured in lateral view as a straight line running from the most ventral to the most dorsal surface of the petiolar node, using tangential lines where required.

PeI Petiole Index: PeL/PeH × 100.

PeL Maximum length of petiole. For workers, alate queens and ergatoid queens the distance from the posterior face to the anterior face of the petiolar node measured in lateral view. For males, the distance from the posterior face of the petiolar node to the junction with the mesosoma measured in lateral view.

PpH Maximum height of the postpetiole measured in lateral view as a straight line running from the most ventral to the most dorsal surface of the postpetiolar node.

PpI Postpetiole Index: PpL/PpH × 100.

PpL Maximum length of the postpetiole measured in lateral view from the posterior face to the anterior face of the postpetiolar node.

PW Maximum width of the pronotum in dorsal view.

SI Scape Index: SL/HW × 100.

SL Length of the antennal scape, including the lamella encircling the base of the scape but excluding the basal condyle.

TL Total length of specimen from tip of mandible to tip of gaster: TL1 + TL2 + TL3 + ML. (Note: The measurements of TL do not include those individuals whose gasters are abnormally prolonged.) TL1: A line measured in lateral view from the apex of the closed mandibles to the midpoint of a straight line across the occipital margin. TL2: A line measured in lateral view from the posteriormost point of postpetiolar dorsum to the tip of the propodeal lobes. TL3: A line measured in lateral view from the anteriormost point to the posteriormost point of gaster.

## Genus diagnosis in Fiji

*Pristomyrmex* workers can be recognized in Fiji by the following characters. Head shape circular to ovoid. Antennal club 3-segmented. Antenna 11-segmented. Antennal sockets surrounded by a raised sharp-edged ridge. Antennal scrobe absent. Anterior clypeal margin armed with three broad and blunt teeth. Mandibles triangular with four large teeth on the masticatory margin and one on the basal margin. Mesosoma with erect hairs present. Propodeum either unarmed, armed with acute angles, or armed with triangular spines distinctly longer than diameter of propodeal spiracle. Waist 2-segmented. Petiole pedunculate; lacking a large anteroventral subpetiolar process. Postpetiole not swollen; in dorsal view not distinctly broader than long or distinctly wider than petiole.

### Pristomyrmex tsujii sp. n.

http://zoobank.org/AC753BF0-6236-48B8-B8D0-72AEC08C9439

http://species-id.net/wiki/Pristomyrmex_tsujii

Figs 1 –6 , 11–22 , 35

Pristomyrmex sp. FJ02. Sarnat and Economo 2012:116, pl. 113 (key, discussion, checklist).

#### Holotype.

Fiji, Lomaiviti Prov., Koro I., 2.7 km NW Nasau Village, footrack b/w Mt. Kuitarua & Nasau, 12.iii.2005, 465m, -17.2947, 179.406, primary rainforest, litter sifting, ex. soil, leaf litter, decaying wood, EMS1862.01, E.M. Sarnat (worker, dry pinned, BPBM, specimen code CASENT0171143). *Paratype*: 1 dealate queen, same data as holotype (USNM, specimen code CASENT0171144).

#### Diagnosis.

*Pristomyrmex tsujii* workers are polished red, stoutly built and often foveolate. The propodeum is either armed with small denticles or entirely unarmed. The lack of strong propodeal spines (Figs 5–6) separates workers, ergatoid queens and alate queens of *Pristomyrmex tsujii* (Figs 12, 13, 18) from those of the sympatric *Pristomyrmex mandibularis* (Figs 24, 27, 30). The same character is used to diagnose the males, but the spines are reduced to denticles in *Pristomyrmex mandibularis* (Fig. 33) and entirely absent in *Pristomyrmex tsujii* (Fig. 21). Additionally, the males of *Pristomyrmex tsujii* tend more towards brown than black. The only congeneric species with an unarmed propodeum is *Pristomyrmex inermis* Wang from New Guinea which also belongs to the *levigatus* group. *Pristomyrmex tsujii* has a more nodiform petiole (Figs 3–4), a stronger median clypeal tooth (Fig. 10), and more abundant foveae between the frontal carinae (Figs 7–10).

**Figures 1–2.**
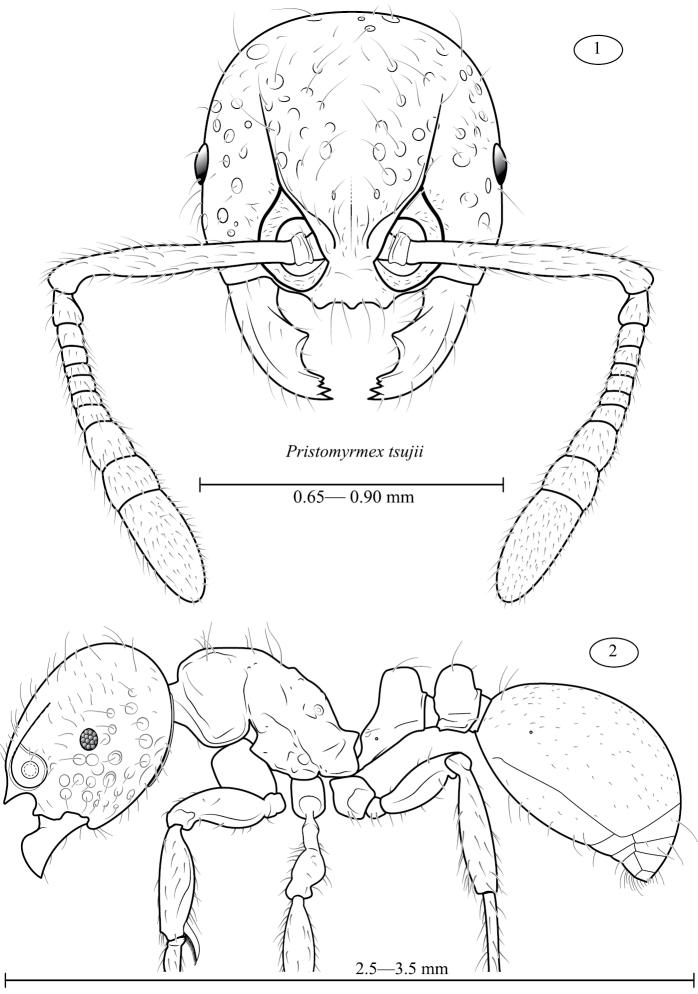
*Pristomyrmex tsujii*, illustrations of head (1) and profile (2). Scale bars are labelled with the approximate observed range for head width (HW) and total length (TL), respectively.

**Figures 3–10.**
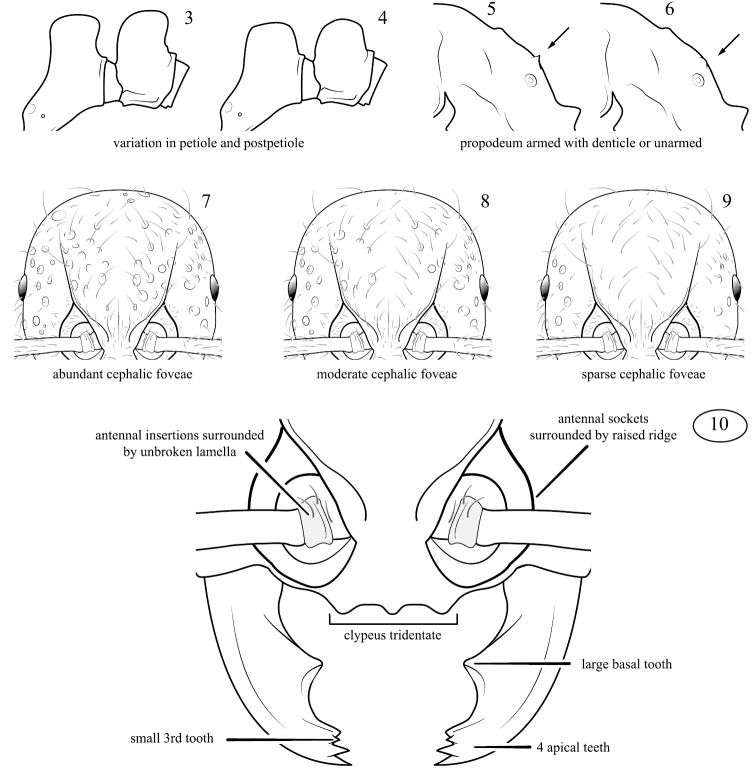
**3–4**.Variation of the petiole and postpetiole observed among populations of *Pristomyrmex tsujii* (**3** Vanua Levu, **4** Koro) **5–6** Variation of the propodeum observed among populations of *Pristomyrmex tsujii* (**5** armed with denticles, **6** unarmed) **7–9** Variation of cephalic sculpture observed among populations of Fijian *Pristomyrmex*. *Pristomyrmex tsujii* tends towards abundant (**7**) to moderate (**8**) cephalic foveae, while *Pristomyrmex mandibularis* varies from abundant (**7**) to sparse (**9**). On Viti Levu, the cephalic sculpture of *Pristomyrmex mandibularis* varies along geographical gradients, whereas on Koro colonies exhibiting the two extremes of the sculpture spectrum occur sympatrically without any known intermediates **10** Taxonomic characters of the mandibles and anterior head capsule used in combination to separate *Pristomyrmex* species from all other Fijian myrmicines.

**Figures 11–22.**
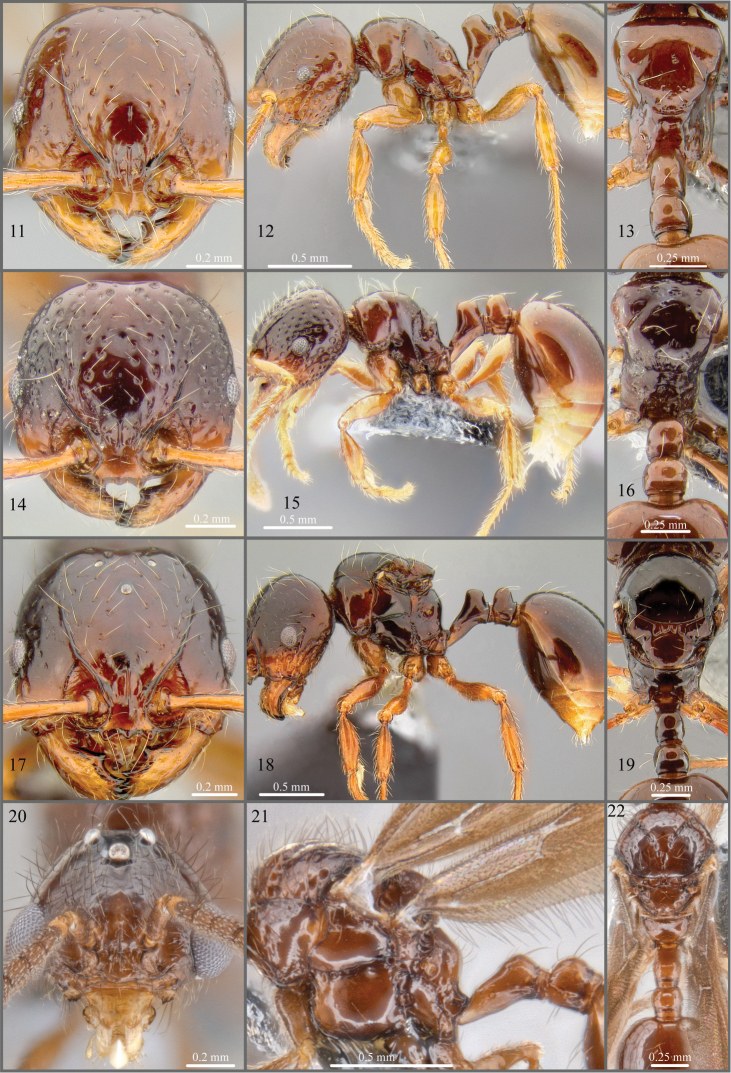
**11–13** *Pristomyrmex tsujii* sp. n., holotype worker, specimen code CASENT0171144. **14–16** *Pristomyrmex tsujii* sp. n., ergatoid queen, specimen code CASENT0181693**17–19** *Pristomyrmex tsujii* sp. n., paratype alate queen, specimen code CASENT0171143**20–22** *Pristomyrmex tsujii* sp. n., male, specimen code CASENT0181883.

**Worker** (Figs 1–6, 11–13). *Measurements* (*n* = 20): TL 2.63–3.35, HW 0.66–0.88, HL 0.67–0.87, CI 96–105, SL 0.59–0.77, SI 84–93, EL 0.08–0.11, PW 0.41–0.6, ML 0.61–0.79, PeH 0.24–0.29, PeL 0.14–0.18, PeI 54–71, PpH 0.25–0.31, PpL 0.14–0.19, PpI 47–67. *Holotype measurements*: TL 3.03, HW 0.79, HL 0.81, CI 99, SL 0.70, SI 89, EL 0.1, PW 0.5, ML 0.72, PeH 0.25, PeL 0.14, PeI 58, PpH 0.25, PpL 0.16, PpI 65.

**Figures 23–34.**
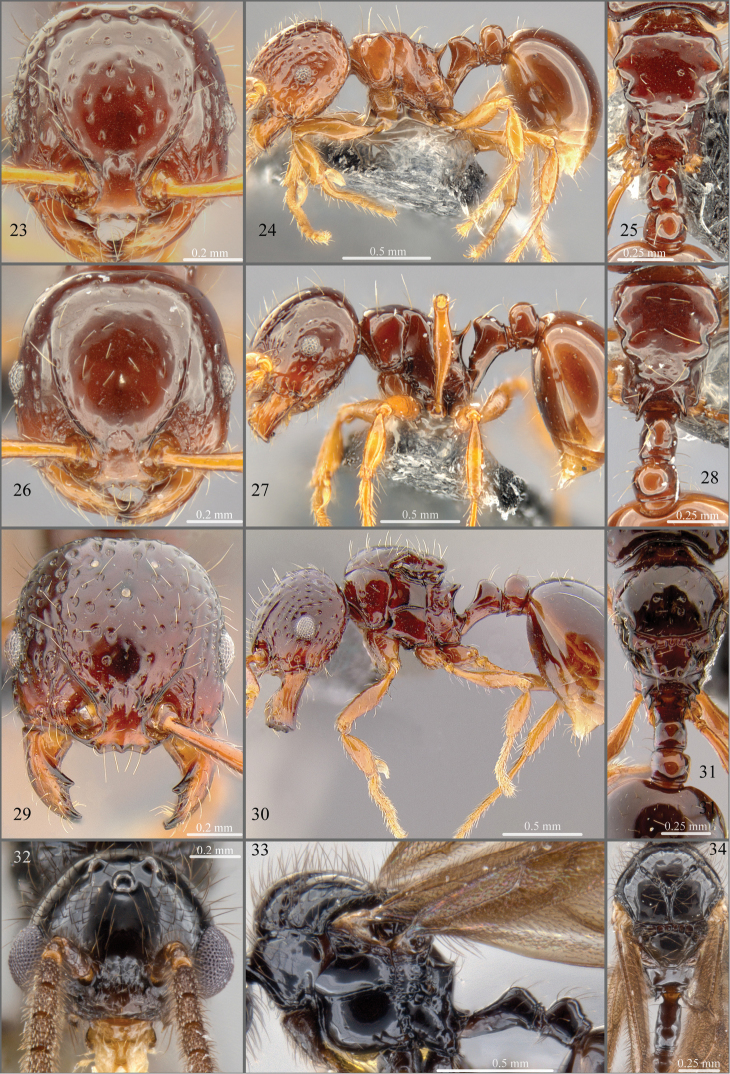
**23–25** *Pristomyrmex mandibularis* Mann, worker, specimen code CASENT0181756. **26–28** *Pristomyrmex mandibularis* Mann, worker, specimen code CASENT0181648 **29–31** *Pristomyrmex mandibularis* Mann, alate queen, specimen code CASENT0171100**32–34** *Pristomyrmex mandibularis* Mann, male, specimen code CASENT0182032.

**Figure 35.**
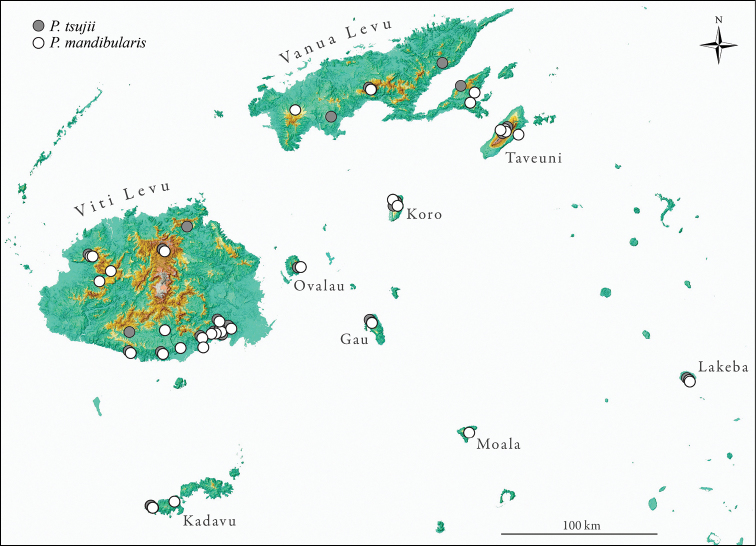
Known distribution of *Pristomyrmex tsujii* (white) *Pristomyrmex mandibularis* (gray) across the Fijian archipelago. Names are given for all islands on which specimens were collected. Symbols have been minimally offset from each other so as not to obscure localities where both species have been recorded.

Conforming to the generic *Pristomyrmex* and *levigatus* species group definitions detailed in Wang (2003) with the following specifications. Head shape circular with posterior margin flat to feebly concave medially in full-face view. Antenna 11-segmented with apical three segments forming a distinct club. Antennal insertion surrounded by a raised and unbroken lamella. Frontal carina distinct and extends just past the level of the posterior eye margin. Weak median carina, approximately same length as terminal antennal segment, extending posteriorly from between antennal insertions and transitioning into a weak median groove that terminates near eye level. Frontal lobe weakly expanded as a thin lamella. Eye moderate-sized, approximately same size as antennal socket, 3–4 facets along longest diameter. Clypeus flat and unsculptured. Median part of clypeus shield-like, projecting posteriorly between the bases of the antennae. Anterior clypeal margin tridentate with a median tooth and two lateral teeth; the median tooth similar in size or slightly smaller than the others. Ventral surface of clypeus smooth, lacking a transverse ruga. Lateral portions of clypeus anterior to antennal insertions reduced to a narrow margin. Mandibles mostly smooth with a few weak striae. Masticatory margin of mandible lacking a diastema and possessing four teeth. The third tooth, counting from the apex, is the smallest. A strongly prominent tooth present about midway on the basal margin of mandible. Anterior portion of dorsal labrum with two tooth-like prominences. Palp formula 1,3. Dorsum of mesosoma in profile view evenly arched, broken only by a weak impression separating the mesonotum from the propodeum. Pronotum unarmed; indistinct obtuse humeral angle. Propodeum armed with pair of small but distinct acute denticles to entirely unarmed. Propodeal lobes triangular, obtusely rounded. Fore tibial spur pectinate. Middle and hind tibiae lacking spurs. Petiole node in profile high, taller than long, with anterior face weakly convex, dorsal face flat to weakly convex, and posterior faces weakly convex to weakly concave. Petiolar peduncle tapering broadly into petiolar node and approximately as long as petiolar node. Postpetiole in profile as tall or occasionally taller than petiole, approximately two times as tall as long; anterior face sloping evenly into dorsal face and junction of posterior face and dorsal face more angular. Dorsum of head covered with scattered to abundant weakly impressed foveae. Dorsum of mesosoma smooth and shining. Petiole and postpetiole smooth and shining, each with a weak lateral longitudinal carina on both sides. Gaster unsculptured. Dorsal surface of head with numerous erect to suberect long hairs originating from center of foveolae. Mesosoma with 4–5 pairs of long erect hairs. Petiolar peduncle with one pair of erect hairs. Petiolar and postpetiolar nodes each with one pair of posteriorly projecting erect hairs. First gastral segment with 1–3 pairs of erect hairs on anterior third. Scape and tibia with numerous erect to suberect hairs. All surfaces a shiny, polished yellowish brown to reddish brown.

**Ergatoid queen** (Figs 14–16). *Measurements* (*n* = 3): TL 3.25, HW 0.85–0.88, HL 0.83–0.84, CI 101–105, SL 0.68–0.73, SI 80–85, EL 0.11–0.12, PW 0.54–0.59, ML 0.75–0.79, PeH 0.26–0.28, PeL 0.16–0.18, PeI 61–66, PpH 0.27–0.30, PpL 0.16–0.19, PpI 55–63.

Closely resembling the worker in the structure of mandibles, clypeus, petiole, postpetiole and gaster in addition to sculpture, color and pilosity. Head with a single well-defined depression in place of the median ocellus. Mesosoma in dorsal view with a promesonotal suture but lacking sclerites associated with alate queen. Mesonotum more convex. Propodeal spines either absent or reduced to acute angles. Dorsum of head covered with scattered to abundant weakly impressed foveolae and smaller shallow punctures. Dorsum of mesosoma similar to alate queen with one or two additional pairs of erect hairs than worker.

**Alate queen** (Figs 20–22). *Measurements* (*n* = 2): TL 3.67–3.94, HW 0.88–0.91, HL 0.86–0.91, CI 100–103, SL 0.75–0.80, SI 85–88, EL 0.16, PW 0.66–0.69, ML 0.95–1.01, PeH 0.27–0.28, PeL 0.16, PeI 58–61, PpH 0.29–0.30, PpL 0.17, PpI 54–58.

Conforming to the generic *Pristomyrmex* definition detailed in Wang (2003) with the following specifications. Closely resembling worker in the structure of the mandibles, clypeus, petiole, postpetiole and gaster in addition to sculpture, color and pilosity with the following differences. Larger. Eyes much larger with diameter composed of ca. 12 facets. Three ocelli present. Posterior head margin weakly concave. Mesosoma marked with wing sclerites and dorsal sutures. Wing shape and venation unknown (only dealate specimens available for examination). Propodeal spines either absent or reduced to acute angles. Dorsum of head covered with scattered to abundant weakly impressed foveolae and smaller shallow punctures. Dorsum of mesosoma with more than 10 pairs of erect hairs.

**Male**. (Figs 23–25). *Measurements* (*n* = 11): TL 2.95–3.41, HW 0.52–0.64, HL 0.46–0.61, CI 103–112, SL 0.14–0.20, SI 24–32, HWE 0.69–0.80, EL 0.24–0.28, PW 0.50–0.73, ML 0.93–1.05, PeH 0.14–0.20, PeL 0.32–0.40, PeI 178–232, PpH 0.17–0.21, PpL 0.11–0.18, PpI 55–76.

Conforming to the generic *Pristomyrmex* definition detailed in Wang (2003) with the following specifications. Head, including the eyes, broader than long. Dorsal portion of occipital margin raised into a transverse carina from which short lengths of longitudinal carinae originate. Frontal carina weak, terminating before reaching the posterior level of the eye. Clypeus with a median longitudinal carina and 1–3 pair of lateral carinae extending towards the anterior margin. Anterior clypeal margin flat to weakly convex. Mesoscutum with distinct notauli forming a Y-shape. Parapsidal furrows reduced to weak impressions. Scuto-scutellar sulcus with 7–10 narrow longitudinal ridges visible in dorsal view. Propodeum unarmed to weakly tuberculate, lacking teeth or spines. Propodeal lobes obtusely triangular with a blunt or rounded apex; sometimes reduced to weak flanges. Middle and hind tibiae lacking spurs. Petiole in profile cuneiform; node with a convex posterior face but lacking a distinct anterior face. Peduncle long. Postpetiole in profile weakly nodiform with a steeply convex anterior face and shorter, more gently sloped posterior face. In dorsal view subrectangular and broader than long. Dorsum of head smooth and shining. Dorsal scutum weakly foveolate. Sides of mesosoma smooth and shining, occasionally with several short carinulae on metapleuron and propodeum. Petiole and postpetiole smooth and shining. Gaster unsculptured. All dorsal surfaces with abundant long hairs. Legs and scapes with numerous erect or suberect short hairs. Color reddish brown with lighter brown appendages. Wings infuscated.

#### Geographic variation.

*Pristomyrmex tsujii* varies in the abundance of the cephalic foveae, propodeal armament and petiolar node shape. In Koro (the type locality) and Gau the specimens exhibit a sparse scattering of foveae and punctures usually separated from each other by a distance exceeding their diameters. None of the Koro specimens are armed even with denticles and the petiolar node is relatively broad in profile with a weakly convex posterior face. The series from Taveuni has sparse fovea and the worker from Vanua Levu has moderate foveae. Workers from both islands have an unarmed propodeum, like those from Koro, but the petiolar node is narrower in profile with a weakly concave posterior face. The postpetiolar nodes of workers from both Taveuni and Vanua Levu are taller than the petiolar node. The workers from Viti Levu are all more foveolate than those from the outlying islands. The strongest sculpture was found on a specimen from Waivudawa (CASENT0181827). The petiolar and postpetiolar shapes of Viti Levu workers are more similar to those of Koro workers than those of Taveuni and Vanua Levu. Some of the Viti Levu workers have an unarmed propodeum like those of the outlying islands, whereas others have a propodeum armed with an acute denticle equal or less than the size of the propodeal lobe.

#### Etymology.

This new species is named for Prof. Kazuki Tsuji in honor of his extensive work on the social biology of *Pristomyrmex punctatus*, and his efforts to promote connectivity between the Japanese and international research communities. The species epithet is a noun in apposition and thus invariant.

#### Discussion.

Despite being widely distributed across the Fijian archipelago, workers of *Pristomyrmex tsujii* were rarely encountered in the field, although males were collected in Malaise traps with some frequency. Workers were collected from Gau, Koro, Vanua Levu and Viti Levu. Of these, all were collected in leaf litter samples except for one found foraging on a fallen tree and another found foraging under a stone. Collection records suggest the species prefers primary rainforest, but several collections from secondary forests and forest fragments suggest it can tolerate some degree of disturbance. The strongly distended gasters of the ergatoid queens are presumably equipped with functional ovaries, but a more thorough examination of fresh material would be required to verify their reproductive potential. It is also unknown whether the ergatoid queens occur in the same nests as alate queens, or if they are capable of founding their own colonies.

#### Material examined.

**FIJI. Gau:** Mt. Delaco, 3 km SE Navukailagi Village, 432m, -17.9795, 179.276 (CASENT0181697); Mt. Delaco, 3.3 km SE Navukailagi Village, 387m, -17.98, 179.275 (CASENT0181847, CASENT0181848, CASENT0181869, CASENT0181871, CASENT0181900, CASENT0181917, CASENT0181927, CASENT0181929, CASENT0181939, CASENT0181941, CASENT0181956, CASENT0181960, CASENT0181971, CASENT0181974, CASENT0181975, CASENT0181980, CASENT0181987, CASENT0181992, CASENT0182077); Mt. Delaco, 3.5 km SE Navukailagi Village, 490m, -17.9827, 179.276 (CASENT0181680); Mt. Delaco, 3.8 km SE Navukailagi Village, 496m, -17.9836, 179.277 (CASENT0181844, CASENT0181855, CASENT0181862, CASENT0181863, CASENT0181864, CASENT0181878, CASENT0181912, CASENT0181923, CASENT0181928, CASENT0181969, CASENT0181986, CASENT0181995); Mt. Delaco, 3.9 km SE Navukailagi Village, 575m, -17.9879, 179.278 (CASENT0181786); Mt. Delaco, 4.0 km SE Navukailagi Village, 564m, -17.9861, 179.277 (CASENT0181889, CASENT0181907, CASENT0181913, CASENT0181935, CASENT0181946, CASENT0181947, CASENT0181951, CASENT0181962, CASENT0181973, CASENT0181996). **Kadavu:** Mt. Washington summit, 1.6 km SW Lomaji, 800m, -19.1181, 177.988 (CASENT0182030); Mt. Washington, 1.3 km SSW Lomaji, 580m, -19.1175, 177.992 (CASENT0182000); Mt. Washington, 1.4km SSW Lomaji Village, 700m, -19.1183, 177.99 (CASENT0182060). **Koro:** 2.7 km NW Nasau Village, footrack b/w Mt. Kuitarua & Nasau, 465m, -17.2947, 179.406 (CASENT0171143, CASENT0171144); Mt. Kuitarua summit, 3.8 km NW Nasau Village, 505m, -17.2868, 179.404 (CASENT0182004, CASENT0182038); Mt. Kuitarua, 3.1 km WNW Nasau Village, 440m, -17.2905, 179.404 (CASENT0194546); Mt. Kuitarua, 3.8 km NW Nasau Village, 485m, -17.2888, 179.404 (CASENT0182037); Mt. Nabukala, 4.7 km WSW Nasau Village, 500m, -17.3122, 179.388 (CASENT0181717). **Lakeba:** 3.2 km NE Tubou Village, 100m, -18.2294, -178.779 (CASENT0182083). **Ovalau:** 1.2 km NNW Draiba Village, 300m, -17.6942, 178.825 (CASENT0181745). **Taveuni:** Devo Peak, 5.5 km SE Tavuki Village, 1188m, -16.8432, -179.966 (CASENT0181898, CASENT0181940); Devo Peak, 5.6 km SE Tavuki Village, 1187m, -16.8433, -179.96 (CASENT0181981); Mt. Devo, 5.3 km SE Tavuki Village, 1064m, -16.8409, -179.968 (CASENT0181865, CASENT0181885, CASENT0181961, CASENT0182007, CASENT0182009); Mt. Devo, Tavuki Village, 892m, -16.8372, -179.973 (CASENT0181915, CASENT0181919, CASENT0194570); Soqulu Estate, 140m, -16.8333, -180 (CASENT0182019). **Vanua Levu:** 1.5 km N Yasawa Village, 300m, -16.4681, 179.644 (CASENT0181708); Batiqere Range, 6 km NW Kilaka Village, 61m, -16.8108, 178.988 (CASENT0194551); Natewa Peninsula, Mt. Navatadoi, hilltop, 2.6 km SSE Vusasivo Village, 400m, -16.5928, 179.772 (CASENT0182031); Vatudiri, 4 km SE Lomaloma Village, 630m, -16.6296, 179.208 (CASENT0182005, CASENT0182022, CASENT0182028). **Viti Levu:** 1.8 km NW Naboutini Village, 300m, -18.2206, 177.817 (CASENT0181734); 2.3 km NW Nabukelevu Village, 300m, -18.11, 177.817 (CASENT0181655, CASENT0181747); 2.7 km NE Naikorokoro Village, 300m, -18.0872, 178.331 (CASENT0181679); 4.8 km NE Galoa Village, 300m, -18.2186, 178.014 (CASENT0181750); 7.5 km NE Vunisea Village, 300m, -17.4833, 178.143 (CASENT0181837); Mt. Evans Range, Koroyanitu Eco Park, 0.5 km N Abaca Village, 800m, -17.6667, 177.55 (CASENT0182006, CASENT0182046, CASENT0182121, CASENT0182128); Mt. Evans Range, Koroyanitu Eco Park, Kokabula Trail, 1 km E Abaca Village, 800m, -17.6667, 177.55 (CASENT0182001, CASENT0182117, CASENT0181894, CASENT0181936, CASENT0182017, CASENT0182024, CASENT0182059, CASENT0182071, CASENT0182075, CASENT0182079, CASENT0182089, CASENT0182123, CASENT0182146); Mt. Korobaba 1.0 km SW Lami Town, 200m, -18.0867, 178.379 (CASENT0181800); Mt. Korobaba near Lami Town, 300m, -18.0167, 178.35 (CASENT0181664, CASENT0181779, CASENT0194572); Mt. Korobaba, 4 km NW Lami Town, 260m, -18.1042, 178.381 (CASENT0182042); Mt. Korobaba, 4 km NW Lami Town, 400m, -18.1022, 178.383 (CASENT0181931); Mt. Nakobalevu, 4 km WSW Colo-i-Suva Village, 325m, -18.0558, 178.422 (CASENT0181950); Mt. Nakobalevu, 4 km WSW Colo-i-Suva Village, 372m, -18.0553, 178.424 (CASENT0181849, CASENT0181881, CASENT0181883, CASENT0181896, CASENT0181899, CASENT0181922, CASENT0181943, CASENT0181985, CASENT0182035, CASENT0182052, CASENT0182138); Mt. Nakobalevu, TV Tower, 5 km WSW Colo-i-Suva Village, 460m, -18.05, 178.417 (CASENT0181909, CASENT0181984, CASENT0182047, CASENT0182140); Mt. Tomanivi, 1.8 km E Navai Village, 700m, -17.6211, 177.998 (CASENT0182141); Mt. Tomanivi, 2 km E Navai Village, 700m, -17.6211, 178 (CASENT0182143); near Nabukavesi Village, 300m, -18.1167, 178.25 (CASENT0181669, CASENT0181693, CASENT0181777, CASENT0181785, CASENT0181791); Waivudawa Creek 6.0 km NNW Lami Town, 300m, -18.076, 178.363 (CASENT0181670, CASENT0181827); Waivudawa, 3.5 km N Veisari Settlement, 300m, -18.0681, 178.367 (CASENT0182013, CASENT0182074, CASENT0182139, CASENT0182002, CASENT0182020, CASENT0182036, CASENT0182126).

### Pristomyrmex mandibularis

Mann

http://species-id.net/wiki/Pristomyrmex_mandibularis

Figs 23 –35

Pristomyrmex mandibularis Mann 1921:444; worker described. Type locality: Fiji, Viti Levu, Nadarivatu, W.M. Mann [examined]. Wang 2003: 505, figs 225–228 (generic revision, ergatoid queen described). Sarnat and Economo 2012: 115, pl. 112 (key, discussion, checklist).

#### Diagnosis.

**Alate queen** (Figs 29–31). *Measurements* (*n* = 10): TL 3.22–3.65, HW 0.80–0.86, HL 0.77–0.85, CI 99–106, SL 0.64–0.74, SI 79–88, EL 0.14–0.18, PW 0.61–0.67, ML 0.82–0.96, PeH 0.22–0.26, PeL 0.14–0.18, PeI 58–76, PpH 0.26–0.29, PpL 0.16–0.19, PpI 59–69. *Measurements of aberrant specimen CASENT0194557*: TL 4.10, HW 1.01, HL 0.96, CI 105, SL 0.77, SI 77, EL 0.19, PW 0.76, ML 1.06, PeH 0.30, PeL 0.17, PeI 58, PpH 0.32, PpL 0.21, PpI 65.

Conforming to the generic *Pristomyrmex* definition detailed in Wang (2003) with the following specifications. Closely resembling worker in the structure of the mandibles, clypeus, petiole, postpetiole and gaster in addition to sculpture, color and pilosity with the following differences. Distinctly larger. Eyes much larger with diameter composed of ca. 12 facets. Three ocelli present. Posterior head margin flat to weakly concave. Median clypeal tooth distinct to nearly absent. Mesosoma marked with wing sclerites and dorsal sutures. Propodeal spines significantly smaller than those of worker; present as large and broad distinct angles approximately equal in size to propodeal lobes. Dorsum of head entirely free of foveae to covered in abundant well-defined foveae. Dorsum of mesosoma with more than 10 pairs of erect hairs.

**Male** (Figs 32–34). *Measurements* (*n* = 21): TL 2.55–3.22, HW 0.51–0.61, HWE 0.63–0.75, HL 0.48–0.6, CI 97–113, SL 0.14–0.21, SI 26–34, EL 0.21–0.25, PW 0.53–0.73, ML 0.8–1.08, PeH 0.14–0.19, PeL 0.27–0.38, PeI 178–234, PpH 0.18–0.23, PpL 0.13–0.17, PpI 64–83.

Conforming to the generic *Pristomyrmex* definition detailed in Wang (2003) with the following specifications. Head, including the eyes, broader than long. Dorsal portion of occipital margin raised into a transverse carina from which no short lengths of longitudinal carinae originate. Frontal carina weak, terminating before reaching the posterior level of the eye. Clypeus with a median longitudinal carina and 1–3 pair of lateral carinae extending towards the anterior margin. Anterior clypeal margin flat to weakly convex. Mesoscutum with distinct notauli forming a Y-shape. Parapsidal furrows reduced to weak impressions. Scuto-scutellar sulcus with 7–10 narrow longitudinal ridges visible in dorsal view. Propodeum armed with a pair of strong tubercles or small teeth. Propodeal lobes obtusely triangular with a blunt or rounded apex; usually more developed than propodeal teeth. Middle and hind tibiae lacking spurs. Petiole in profile cuneiform with a weakly developed and broadly convex node. Peduncle long. Postpetiole in profile nodiform with a steeply convex anterior face and shorter, more gently sloped posterior face. In dorsal view subrectangular and broader than long. Entirely smooth and shiny. All dorsal surfaces with abundant long erect to suberect hairs. Legs and scapes with numerous erect or suberect short hairs. Color black to blackish brown with lighter brown appendages and gaster. Wings infuscated.

#### Geographic variation.

Like the worker of the species, which is discussed in Wang (2003) and Sarnat and Economo (2012), the alate queens of *Pristomyrmex mandibularis* vary substantially in size, shape, color and sculpture across the archipelago. The Viti Levu specimens are marked by sparse foveae, a median clypeal tooth that is smaller than the lateral teeth but still distinct, and tend towards the redder end of the color spectrum. The Vanua Levu, Koro and Lakeba specimens are moderately foveolate with a smaller and more blunted median clypeal tooth.

#### Comments.

A puzzling taxonomic situation is presented by two alate queens from Gau occupying the extreme ends of the sculpture spectrum. Specimen CASENT0181910 lacks any distinct foveae on the head and mesosoma and is the least sculptured of the examined queens. Specimen CASENT0194557 is both exceptionally large (HW = 1.01 mm) and the mostly strongly sculptured of all examined Fijian *Pristomyrmex* queens, with well-defined foveae covering the head. The abundant pilosity on the head and mesosoma can be perhaps be explained by the greater number of piligerous foveae, but the length of the hairs is distinctly shorter than all other alate queens, including the aforementioned CASENT0181910 from Gau.

**Figure 36.**
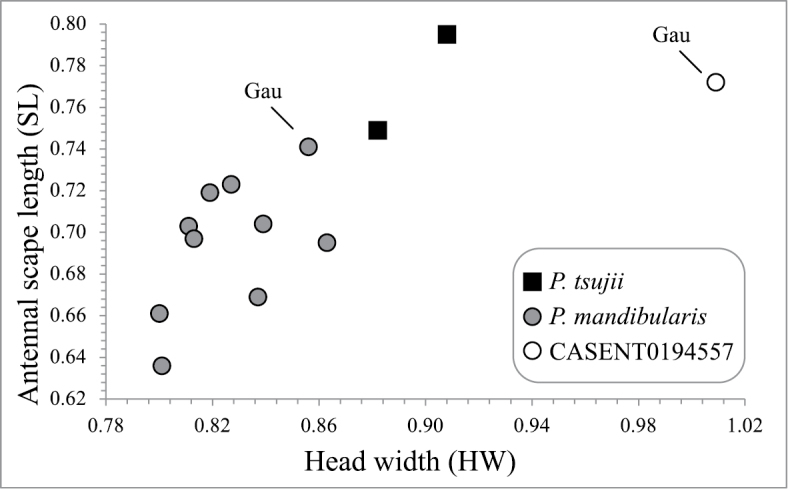
Antennal scape length (SL) *versus* head width (HW) for alate queens of *Pristomyrmex mandibularis* and *Pristomyrmex tsujii*. Circles represent *Pristomyrmex mandibularis*, squares represent *Pristomyrmex tsujii*. The white circles represent the aberrant specimen CASENT0194557 from Gau.

Specimen CASENT0194557 also has shorter antennal scapes relative to its head width than any other examined alate queens of *Pristomyrmex mandibularis* or *Pristomyrmex tsujii* (Fig. 36). The combination of gross morphological differences, morphometric disparity and sympatry of the two alate queens from Gau suggests that CASENT0194557 be assigned to a different species. We are reluctant however to describe a new species based on a single queen specimen. The aberrant measurements of CASENT0194557 are therefore reported separately from those collated from all other *Pristomyrmex mandibularis* alate queens.

#### Material examined.

**FIJI. Gau:** Mt. Delaco, 3 km SE Navukailagi Village, 408m, -17.9796, 179.276 (CASENT0181652, CASENT0181752, CASENT0181761); Mt. Delaco, 3 km SE Navukailagi Village, 432m, -17.9795, 179.276 (CASENT0181751); Mt. Delaco, 3.3 km SE Navukailagi Village, 387m, -17.98, 179.275 (CASENT0182033, CASENT0182065, CASENT0182090, CASENT0182092, CASENT0182093, CASENT0182098, CASENT0194557); Mt. Delaco, 3.4 km SE Navukailagi Village, 475m, -17.9836, 179.277 (CASENT0181699); Mt. Delaco, 3.5 km SE Navukailagi Village, 490m, -17.9827, 179.276 (CASENT0181672); Mt. Delaco, 3.8 km SE Navukailagi Village, 496m, -17.9836, 179.277 (CASENT0181910, CASENT0182016, CASENT0182021, CASENT0182032, CASENT0182051, CASENT0182054, CASENT0182062, CASENT0182078, CASENT0182091); Mt. Delaco, 4.0 km SE Navukailagi Village, 564m, -17.9861, 179.277 (CASENT0182034); Mt. Delaco, 4.4 km SE Navukailagi Village, 625m, -17.9879, 179.278 (CASENT0181644); 2.5 km SE Navukailagi Village, 300m, -17.9714, 179.272 (CASENT0181816). **Kadavu:** Moanakaka Bird Sanctuary, 0.25km SW Soladamu Village, 60m, -19.0775, 178.121 (CASENT0181597);, CASENT0182044, CASENT0182049, CASENT0182053, CASENT0182055, CASENT0182085, CASENT0182087, CASENT0182094, CASENT0182096, CASENT0182097); Mt. Washington summit, 1.6 km SW Lomaji, 800m, -19.1181, 177.988 (CASENT0182025); Mt. Washington, 1.3 km SSW Lomaji, 580m, -19.1175, 177.992 (CASENT0182069); Mt. Washington, 1.4km SSW Lomaji Village, 700m, -19.1183, 177.99 (CASENT0181796); Mt. Washington, 1.4km SSW Lomaji Village, 760m, -19.1181, 177.988 (CASENT0181712). **Koro:** 1.6 km E Tavua Village, 220m, -17.2753, 179.374 (CASENT0181645, CASENT0181782, CASENT0181808, CASENT0181831); 2.1 km SW Nabuna Village, nr. Wailolo Creek, 115m, -17.2658, 179.37 (CASENT0171044, CASENT0171100, CASENT0181765, CASENT0194552, CASENT0194559, CASENT0194575); 2.7 km NW Nasau Village, footrack b/w Mt. Kuitarua & Nasau, 465m, -17.2947, 179.406 (CASENT0181654); 3.0 km WNW Nasau Village, footrack b/w Mt. Kuitarua & Nasau, 420m, -17.2903, 179.405 (CASENT0171142); Mt. Kuitarua summit, 3.8 km NW Nasau Village, 500m, -17.2875, 179.402 (CASENT0182026, CASENT0182058, CASENT0182061, CASENT0182072, CASENT0182080); Mt. Kuitarua summit, 3.8 km NW Nasau Village, 505m, -17.2868, 179.404 (CASENT0182084, CASENT0182095); 2.0 km N Nasoqoloa Village, 300m, -17.2722, 179.389 (CASENT0181716, CASENT0181718, CASENT0181769, CASENT0194562); Mt. Kuitarua, 4 km WNW Nasau Village, 380m, -17.3, 179.4 (CASENT0181688). **Lakeba:** 3.2 km NE Tubou Village, 100m, -18.2208, -178.784 (CASENT0182063, CASENT0194549, CASENT0182039, CASENT0182068, CASENT0182088)., CASENT0194569). **Moala:** Mt. Korolevu, 5.5 km SW of Naroi Village, 300m, -18.5948, 179.9 (CASENT0182307). **Ovalau:** (coordinates for Levuka), m, -17.68, 178.83 (CASENT0233996). **Taveuni:** Mt. Devo, 3.6 km SE Tavuki Village, 734m, -16.8306, -179.974 (CASENT0181668); Mt. Devo, 3.9 km SE Tavuki Village, 775m, -16.8328, -179.973 (CASENT0181813); Mt. Devo, 5.3 km SE Tavuki Village, 1064m, -16.8409, -179.968 (CASENT0182043); Mt. Koronibuabua, 3.2 km NW Lavena Village, 234m, -16.8547, -179.891 (CASENT0182023, CASENT0182086). Soqulu Estate, 140m, -16.8333, -180 (CASENT0182018). **Vanua Levu:** 2.0 km NW Nakanakana Village, 300m, -16.62, 179.833 (CASENT0181756); Lasema, m, -16.68, 179.81 (CASENT0233997); Mt. Vatudiri, 3 km NW Waisali Village, 570m, -16.629, 179.211 (CASENT0194550); Mt. Vatudiri, 3 km NW Waisali Village, 641m, -16.6285, 179.208 (CASENT0181637); Navotuvotu Range, Mt. Koroimari, logging road nr. Banikea Village, 398m, -16.7675, 178.757 (CASENT0182082); Vatudiri, 4 km SE Lomaloma Village, 630m, -16.6296, 179.208 (CASENT0182040, CASENT0182056, CASENT0182067, CASENT0182070, CASENT0182081). **Viti Levu:** 1.3 km SW Vaturu Dam, 530m, -17.7478, 177.677 (CASENT0182012); 1.5 km NE Colo-i-Suva Village, 340m, -18.0506, 178.417 (CASENT0181724, CASENT0181729, CASENT0194548); 1.8 km NW Naboutini Village, 300m, -18.2206, 177.817 (CASENT0181702); 2.7 km NE Naikorokoro Village, 300m, -18.0872, 178.331 (CASENT0181687, CASENT0181836, CASENT0194934); 4.8 km NE Galoa Village, 300m, -18.2186, 178.014 (CASENT0181659); Koroyanitu Eco Park 5.0 km NE Abaca Village, 700m, -17.6667, 177.553 (CASENT0181662, CASENT0181673, CASENT0181789, CASENT0181790); Mt. Evans Range, Koroyanitu Eco Park, 0.5 km N Abaca Village, 800m, -17.6667, 177.55 (CASENT0182014); Mt. Evans Range, Koroyanitu Eco Park, 1.8 km NE Abaca Village, 700m, -17.6667, 177.563 (CASENT0181773); Mt. Evans Range, Koroyanitu Eco Park, Kokabula Trail, 1 km E Abaca Village, 800m, -17.6667, 177.55 (CASENT0182011, CASENT0182045, CASENT0182147, CASENT0181870, CASENT0181989); Mt. Korobaba near Lami Town, 300m, -18.0167, 178.35 (CASENT0181648, CASENT0181740, CASENT0181807, CASENT0181811, CASENT0181829); Mt. Nakobalevu, 4 km WSW Colo-i-Suva Village, 372m, -18.0553, 178.424 (CASENT0181861); Mt. Rama 1.8 km NW Naikorokoro Settlement, 300m, -18.09, 178.304 (CASENT0181663, CASENT0181759); Mt. Tomanivi, 2 km E Navai Village, 700m, -17.6211, 178 (CASENT0182066); Nasoqo, m, -18.08, 178.02 (CASENT0233995); Nausori Highlands, 400m, -17.81, 177.61 (CASENT0234108); near Nabukavesi Village, 300m, -18.1167, 178.25 (CASENT0181397, CASENT0181778, CASENT0181801); Ocean Pacific Resort, 2 km SE Nabukavesi Village, 40m, -18.1708, 178.258 (CASENT0182064); Waivudawa Creek 6.0 km NNW Lami Town, 300m, -18.076, 178.363 (CASENT0181555); Waiyanitu, m, -18.18, 178.12 (CASENT0233994).

## Supplementary Material

XML Treatment for Pristomyrmex tsujii

XML Treatment for Pristomyrmex mandibularis

## References

[B1] André E (1905) Description d’un genre nouveau et de deux espèces nouvelles de fourmis d’Australie. Revue d’Entomologie (Caen) 24: 205-208.

[B2] Bolton B (2013) An online catalog of the ants of the world. http://antcat.org. [accessed 7 Aug. 2013]

[B3] Brown WL Jr. Wilson EO (1956) Character displacement. Systematic Zoology 5: 49-64. doi: 10.2307/2411924

[B4] Buschinger A Heinze J (1992) Polymorphism of female reproductives in ants. In: Billen JPJ (Ed) Biology and evolution of social insects. Leuven University Press, Leuven ix + 390 p., 11-23.

[B5] Economo EP Sarnat EM (2012) Revisiting the ants of Melanesia and the taxon cycle: historical and human-mediated invasions of a tropical archipelago. American Naturalist 180: E1–E16. doi: 10.1086/665996 22673659

[B6] Evenhuis NL Bickel DJ (2005) The NSF-Fiji terrestrial arthropod survey: overview. Occassional Papers of the Bernice P Bishop Museum 82: 3-25.

[B7] Emery C (1887) Catalogo delle formiche esistenti nelle collezioni del Museo Civico di Genova. Parte terza. Formiche della regione Indo-Malese e dell’Australia (continuazione e fine). [part]. Annali del Museo Civico di Storia Naturale 25[=(2)5]: 433–448.

[B8] Heinze J (1998) Intercastes, intermorphs, and ergatoid queens: who is who in ant reproduction? Insectes Sociaux 45: 113–124. doi: 10.1007/s000400050073

[B9] Heinze J Tsuji K (1995) Ant reproductive strategies. Researches on Population Ecology Kyoto 37: 135-149. doi: 10.1007/BF02515814

[B10] Karavaiev V (1931) Ameisen aus Englisch-Ostafrika. Zoologischer Anzeiger 95: 42-51.

[B11] Lucky A Sarnat EM (2008) New species of *Lordomyrma* (Hymenoptera: Formicidae) from Southeast Asia and Fiji. Zootaxa 1681: 37-46.

[B12] Lucky A Sarnat EM (2010) Biogeography and diversification of the Pacific ant genus *Lordomyrma* Emery. Journal of Biogeography 37: 624-634. doi: 10.1111/j.1365-2699.2009.02242.x

[B13] Mann WM (1921) The ants of the Fiji Islands. Bulletin of the Museum of Comparative Zoology 64: 401-499.

[B14] Mayr G (1866) Diagnosen neuer und wenig gekannter Formiciden. Verhandlungen der Kaiserlich-Königlichen Zoologisch-Botanischen Gesellschaft in Wien 16: 885–908, Tafel XX.

[B15] Peeters C (1991) Ergatoid queens and intercastes in ants: two distinct adult forms which look morphologically intermediate between workers and winged queens. Insectes Sociaux 38: 1–15. doi: 10.1007/BF01242708

[B16] Peeters C (2012) Convergent evolution of wingless reproductives across all subfamilies of ants, and sporadic loss of winged queens (Hymenoptera: Formicidae). Myrmecological News 16: 75-91.

[B17] Sarnat EM (2006) *Lordomyrma* (Hymenoptera: Formicidae) of the Fiji Islands. In: Evenhuis NL Bickel DJ (Eds) Fiji Arthropods VI, Bishop Museum Occasional Papers. Bishop Museum, Honolulu, Hawaii, 9–42, erratum.

[B18] Sarnat EM (2008) A taxonomic revision of the *Pheidole roosevelti*-group (Hymenoptera: Formicidae) in Fiji. Zootaxa 1767: 1-36.

[B19] Sarnat EM Economo EP (2012) Ants of Fiji. University of California Publications in Entomology 132: 1-398.

[B20] Sarnat EM Moreau CS (2011) Biogeography and morphological evolution in a Pacific island ant radiation. Molecular Ecology 20: 114-130. doi: 10.1111/j.1365-294X.2010.04916.x 21059129

[B21] Taylor RW (1965) The Australian ants of the genus *Pristomyrmex*, with a case of apparent character displacement. Psyche (Cambridge) 72: 35-54. doi: 10.1155/1965/74834

[B22] Terayama M (2009) A synopsis of the family Formicidae of Taiwan (Insecta: Hymenoptera). Research Bulletin of Kanto Gakuen University Liberal Arts 17: 81-266.

[B23] Tsuji K (1988) Obligate parthenogenesis and reproductive division of labor in the Japanese queenless ant *Pristomyrmex pungens*. Comparison of intranidal and extranidal workers. Behavioral Ecology and Sociobiology 23: 247-255. doi: 10.1007/BF00302947

[B24] Wang M (2003) A monographic revision of the ant genus *Pristomyrmex* (Hymenoptera: Formicidae). Bulletin of the Museum of Comparative Zoology 157: 383-542.

[B25] Zettel H (2006) On the ants (Hymenoptera: Formicidae) of the Philippine Islands: I. The genus *Pristomyrmex* Mayr, 1866. Myrmecologische Nachrichten 8: 59-68.

